# Enhanced Dentin-Like Mineralized Tissue Formation by AdShh-Transfected Human Dental Pulp Cells and Porous Calcium Phosphate Cement

**DOI:** 10.1371/journal.pone.0062645

**Published:** 2013-05-10

**Authors:** Lunguo Xia, Maolin Zhang, Qing Chang, Lizhen Wang, Deliang Zeng, Xiuli Zhang, Zhiyuan Zhang, Xinquan Jiang

**Affiliations:** 1 Department of Oral and Maxillofacial Surgery, Shanghai Ninth People's Hospital Affiliated to Shanghai Jiao Tong University, School of Medicine, Shanghai, China; 2 Oral Bioengineering Lab, Shanghai Research Institute of Stomatology, Shanghai Ninth People's Hospital Affiliated to Shanghai Jiao Tong University, School of Medicine, Shanghai Key Laboratory of Stomatology, Shanghai, China; 3 Department of Prosthodontics, Shanghai Ninth People's Hospital Affiliated to Shanghai Jiao Tong University, School of Medicine, Shanghai, China; 4 Department of Oral Pathology, Shanghai Ninth People's Hospital Affiliated to Shanghai Jiao Tong University, School of Medicine, Shanghai, China; National University of Ireland, Galway, Ireland

## Abstract

The aim of the present study was to investigate the effect of Sonic hedgehog (Shh) on human dental pulp cells (hDPCs) and the potential of complexes with Shh gene modified hDPCs and porous calcium phosphate cement (CPC) for mineralized tissue formation. hDPCs were cultured and transfected with adenoviral mediated human Shh gene (AdShh). Overexpression of Shh and cell proliferation was tested by real-time PCR analysis, western blotting analysis, and MTT analysis, respectively. The odontoblastic differentiation was assessed by alkaline phosphatase (ALP) activity and real-time PCR analysis on markers of Patched-1 (Ptc-1), Smoothened (Smo), Gli 1, Gli 2, Gli 3, osteocalcin (OCN), dentin matrix protein-1 (DMP-1), and dentin sialophosphoprotein (DSPP). Finally, AdShh-transfected hDPCs were combined with porous CPC and placed subcutaneously in nude mice for 8 and 12 weeks, while AdEGFP-transfected and untransfected hDPCs were treated as control groups. Results indicated that Shh could promote proliferation and odontoblastic differentiation of hDPCs, while Shh/Gli 1 signaling pathway played a key role in this process. Importantly, more mineralized tissue formation was observed in combination with AdShh transfected hDPCs and porous CPC, moreover, the mineralized tissue exhibited dentin-like features such as structures similar to dentin-pulp complex and the positive staining for DSPP protein similar to the tooth tissue. These results suggested that the constructs with AdShh-transfected hDPCs and porous CPC might be a better alternative for dental tissue regeneration.

## Introduction

Tissue engineering technology provides an approach to achieve dentin regeneration to potentially replace or repair the impaired dental tissues caused by caries, trauma, or clinical errors [1]–[2]. This procedure can be defined as the new dental structures including dentin, root structures, and the dentin-pulp complex, are constructed in vitro or in vivo by using scaffolds, cells, and growth factors, alone or in combination, based on the basic principles of biology and engineering [3]–[4].

Dental pulp cells (DPCs) derived from dental pulp contain ectodermic and mesenchymal components, such as neural crest-derived mesenchymal progenitors with plasticity and multipotential capability. It has been shown that DPCs had the potential to differentiate into odontoblasts under the appropriate conditions [5]–[6]. Previous studies have demonstrated that DPCs could be a suitable cell source for dental tissue regeneration; however, no sufficient mineralized tissue formation for DPCs combination with scaffolds alone might limit further clinical application [7].

To enhance the efficacy of DPCs application for dental tissue regeneration, it is important to replicate the permissive signals that induct terminal events in odontoblastic differentiation during tooth development [6]. Among these signals, Sonic hedgehog (Shh) was required at multiple stages of tooth development, such as early tooth initiation and later morphogenesis. It was also demonstrated that Shh was required continuously during odontogenesis [8]–[11]. These studies indicated that Shh had multiple functions in tooth development; however, whether Shh could enhance proliferation and odontoblastic differentiation of human DPCs (hDPCs), and consequently promote mineralized tissue formation was largely unknown.

An appropriate scaffold is also important for dental tissue regeneration. Although several scaffold materials have been reported in previous studies, most of them lacked the property of inducing odontogenesis, which resulted in incomplete tooth tissue regeneration [12]. Calcium phosphate cement (CPC) possessed self-setting, moderate compressive strength even in loading sites and highly biocompatibility properties. It has been extensively studied and clinically used as bone substitute materials [13]–[15]. Recently, it was explored that CPC could facilitate the growth and differentiation of hDPCs in vitro; however, the potential of complexes with hDPCs and CPC for mineralized tissue formation need to be systematically investigated in vivo [16].

In present study, we hypothesized that the complexes with Shh gene modified DPCs and CPC could be used for dental tissue regeneration. In order to verify our hypothesis, adenoviruses containing human Shh gene (AdShh) were constructed and transfected hDPCs, and then, the proliferation and odontoblastic differentiation of AdShh-transfected hDPCs as well as underlying signal pathway mechanism, was investigated for the first time. Finally, AdShh-transfected hDPCs were loaded onto porous calcium phosphate cement (CPC) scaffold, and then implanted in nude mice. The mineralized tissue formation was evaluated by histological and immunohistochemistry analyses.

## Materials and Methods

### Ethics statement

Human samples (extracted upper third molars) were collected at Shanghai Ninth People's Hospital Affiliated to Shanghai Jiao Tong University, School of Medicine. Written informed consent of all human subjects who participated in the experimental investigation was obtained. The study and the consent procedure were approved by Ethical Committees of Shanghai Ninth People's Hospital Affiliated to Shanghai Jiao Tong University, School of Medicine, while all animal experiments were approved by Ethical Committees for Animal Research of Shanghai Ninth People's Hospital Affiliated to Shanghai Jiao Tong University, School of Medicine (Protocol Number: 201301).

### Cells culture and gene transduction

hDPCs were prepared from periodontally healthy and non-carious upper third molars as described previously [17]. After the first passage, hDPCs were cultured in osteogenic medium (DMEM, 10% FBS, 50 µg/mL L-ascorbic acid, 10 mM glycerophosphate and 100 nM dexamethasone). In present study, the cells at passage 4 were used for subsequent experiments.

Adenoviruses containing human Shh gene and enhanced green fluorescent protein (EGFP) gene (AdShh) or control EGFP (AdEGFP) were constructed by Vector Gene Technology Company Limited (VGTC, China). hDPCs were cultured for 24 hours to reach 80% confluence and transduced with AdShh or AdEGFP at a MOI (pfu/cell) of 50, respectively, and then, infected cells were maintained in differentiation medium, Gene transfer efficiency was assessed at 72 hours after gene transduction under a fluorescent microscopy (Leica, Germany) by calculating the percentage of EGFP-expressing cells among all the cells observed, as described previously [18].

After hDPCs were transduced with AdEGFP, and AdShh, the overexpression of Shh was detected on days 3, 6, 9, 12, 15, and 18 using real-time-PCR and western blotting analyses (n = 3 for each group). Briefly, total RNA was isolated using Trizol reagent (Invitrogen, USA) and synthesized complementary DNA (cDNA) with the PrimeScript 1st Strand cDNA Synthesis kit (TaKaRa, Japan), and then, the gene expression of Shh was measured with a real-time PCR system (Bio-Rad, USA), while glyceraldehyde-3- phosphatedehydrogenase (GAPDH) was used as the housekeeping gene for normalization (table S1). As for western blotting analysis, the cells transduced with AdEGFP, and AdShh were collected and lysed with a protein extraction regent containing protease inhibitor cocktail, phosphotase inhibitor cocktail and phenylmethanesulfonyl fluoride (PMSF) (Kangchen, China). Equal amount of protein samples were separated on SDS–polyacrylamide gel electrophoresis (PAGE), and then electro-transferred to a polyvinylidene difluoride (PVDF, Pall, USA) membrane. The membranes were blocked and incubated with rabbit anti human Shh (CST, USA, dilution, 1∶1000), and mouse anti human actin primary antibody (Sigma, USA, dilution, 1∶5000). Finally, the membranes were visualized with horseradish peroxidase (HRP)-conjugated goat anti-rabbit, and rabbit anti-mouse (Beyotime, China) using the ECL plus reagents (Amersham Pharmacia Biotech, USA) by an UVItec ALLIANCE 4.7 gel imaging system. The relative integrated density of each protein band was analyzed by NIH image J 1.64 s, and then normalized to that of the actin. All experiments were performed in triplicate.

### Cell proliferation analysis

The cell proliferation of AdShh-transfected, AdEGFP-transfected or untransfected hDPCs was evaluated by MTT analysis at days 1, 3, 5, and 7 as described in previous study (n = 3 for each group) [19]. Briefly, MTT (Amresco, USA) solution was added, and then incubated for 4 h. Finally, the medium was replaced with dimethyl sulfoxide (DMSO, USA) and the absorbance was measured at 490 nm by ELX Ultra Microplate Reader (Bio-tek, USA). All experiments were performed in triplicate.

### Cell differentiation analysis

At day 14 after hPDCs were transfected with AdShh, ALP staining was performed according to the manufacturer's instructions (Beyotime, China). More importantly, ALP activity was measured at days 7, and 14 after gene transduction following our previous study (n = 3 for each group) [20]. Briefly, the cells were suspended in lysis buffer with 0.2% NP-40. ALP activity was determined by absorbance at 405 nm using p-nitrophenyl phosphate (pNPP, Sigma, USA) as the substrate. Total cellular protein content was determined by the method in aliquots of the same samples with the Bio-Rad protein assay kit, read at 630 nm and calculated according to a series of BSA (Sigma, USA) standards. Finally, ALP activity was expressed as absorbance at 405 nm (OD value), which was normalized to the total cellular proteins. All experiments were performed in triplicate.

Total RNA was isolated and synthesized cDNA (cDNA) at days 3, 6, and 9 after hDPCs transduced with AdShh as described previously (n = 3 for each group) [21]. Real-time PCR analysis was performed on Patched-1 (Ptc-1), Smoothened (Smo), Gli 1, Gli 2, Gli 3, osteocalcin (OCN), dentin matrix protein-1 (DMP-1), and dentin sialophosphoprotein (DSPP) (table S1). All experiments were performed in triplicate.

### Cyclopamine treatment analysis

After transduced with AdShh, hPDCs were treated with Shh signaling pathway inhibitor Cyclopamine (Cyc, Sigma, USA) with a final concentration of 10 µM for 3, 6, and 9 days, and then, total RNA was isolated and synthesized cDNA, respectively. Finally, real-time PCR was performed on OCN, DMP-1, and DSPP as specified previously. Meanwhile, AdShh-transfected and untransfected hDPCs without Cyc treatment were treated as control groups (n = 3 for each group). All experiments were performed in triplicate.

### Preparation of cell material complexes

AdShh-transfected, AdEGFP-transfected and untransfected hDPCs were separately collected and resuspended in FBS free medium with a final concentration of 2×10^7^ cells/mL at day 3, and then, 20 µL cell suspension was added to each porous CPC implant (Φ4 mm×2 mm) (Rebone, China) for the following study in vivo. Besides, the extra AdShh-transfected and untransfected hDPCs/CPC complexes were cultured for 1, and 4 days, fixed in 2% glutaraldehyde for 2 h, and then dehydrated by increasing the concentration of ethanol. Finally, the samples were dried by hexamethyldisilazane, sputter-coated with gold and observed by scanning electron microscopy (SEM) (JEOL, Japan).

### Ectopic mineralized tissue formation analysis

Twelve athymic nude mice were anesthetized by pentobarbital (Nembutal 3.5 mg/100 g) via intramuscular injection after light ether inhalation. Four subcutaneous pockets were created on the back of each mouse with a distance of more than 3 cm between each pocket, which randomly received the following the implants of four groups: group A, CPC alone (n = 6); group B, untransfected hDPCs/CPC complexes (n = 6); group C, AdEGFP-transfectd hDPCs/CPC complexes (n = 6); and group D, AdShh-transfected hDPCs/CPC complexes (n = 6). These mice were housed in temperature- and light-controlled environmental conditions with a 12-hour light and dark cycle, and permitted ad libitum consumption of water and standard pellet chow. Six mice were euthanized by cervical dislocation (CD) at 8 and 12 weeks after implantation, respectively. The implants were harvested, decalcified in 10% EDTA, cross-sectioned along the maximum surfaces of the samples, and stained with hematoxylin-eosin (HE). Finally, mineralized tissue formation area (the percentage of mineralized tissue area among the whole implant), was determined using the average value of the 3 parallel slices selected from each of the 3 equally divided paraffin parts along the cross-section with Image Pro 5.0 system (Media Cybernetics, USA). The mean value of the 3 measurements was calculated for each implant and used to calculate mean value for each group, as described in our previous study [21]

### Immunohistochemistry analysis

Immunohistochemistry analysis for DSPP protein was carried out on the samples of untransfected hDPCs/CPC complexes (group B), AdEGFP-transfectd hDPCs/CPC complexes (group C), and AdShh-transfected hDPCs/CPC complexes (group D) at 12 week time point. Moreover, the samples of human bone and tooth tissues were also treated as control groups. Briefly, tissue slides were deparaffinized and rehydrated, then submerged in H_2_O_2_ for peroxidase quenching. The slides were incubated with primary antibody to DSPP (Abcam, USA, 1∶200 dilution) overnight at 4°C, respectively, after blocking with 3% BSA (Sigma, USA). Finally, HRP-conjugated secondary antibody was applied to the slides for 1 h at room temperature; moreover, diaminobenzidine (DAB) kit was used to develop the color and the slides counterstained with hematoxylin.

### Statistical analysis

All data were reported as mean ± standard deviation. Statistical analysis for the above assays was performed by ANOVA and SNK post hoc using the SAS 8.2 statistical software package (SAS, USA). Values of *p*<0.05 was considered statistically significant.

## Results

### Cell transduction and proliferation analysis

**Figure 1:**
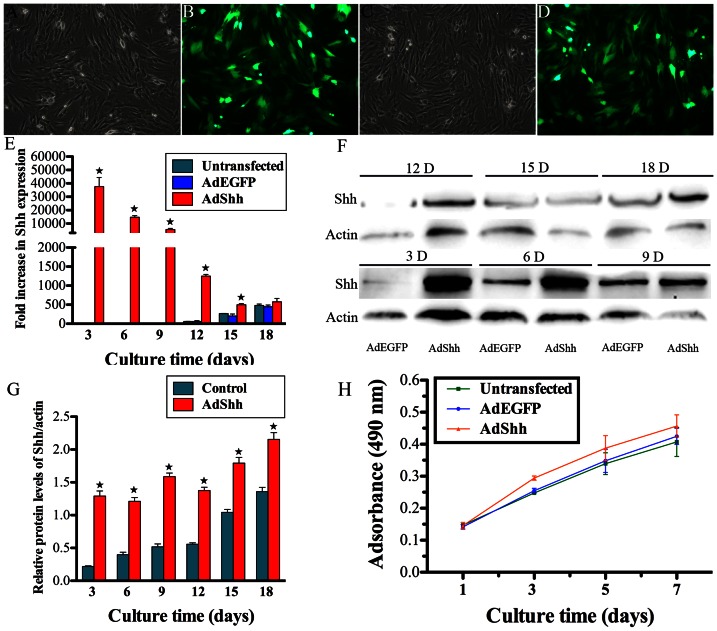
Gene transfer efficiency and cell proliferation. At 72 h after gene transduction, both AdEGFP-transfected (A, B) and AdShh-transfected (C, D) hDPCs grew well and emitted bright and intense green fluorescence. (E–G) Shh mRNA (E) and protein expression (F, G) of hDPCs transduced with AdShh at days 3, 6, 9, 12, 15, and 18. (H) MTT assay of untransfected, AdEGFP-transfected and AdShh-transfected hDPCs at days 1, 3, 5, and 7 for cell viability and proliferation. (A, B, C, D, ×100) (n = 3 for each group, ^★^indicates significant differences between AdShh-transfected vs. untransfected and AdEGFP-transfected, *p*<0.05).

hDPCs grew well and showed strong green fluorescence at 72 hours after either AdEGFP or AdShh transduction, with transfer efficiency of 60–80% (fig. 1A, B, C, D). As shown in fig. 1E, Shh mRNA in AdShh-transfected hDPCs was upregulated to a peak at day 3, then, it gradually decreased with significant difference until 15 days after gene transduction (*p*<0.05). Western blotting analysis demonstrated that significant increase of Shh protein expression in AdShh-transfected hDPCs was detected from 3 days to 18 days, as compared with control group (*p*<0.05) (fig. 1F, G). The growth curve demonstrated enhanced cell proliferation in AdShh-transfected hDPCs compared with either AdEGFP-transfected or untransfected cells (*p*<0.05), while there was no significant difference between AdEGFP-transfected and untransfected cells (fig. 1H).

### Cell differentiation analysis

**Figure 2:**
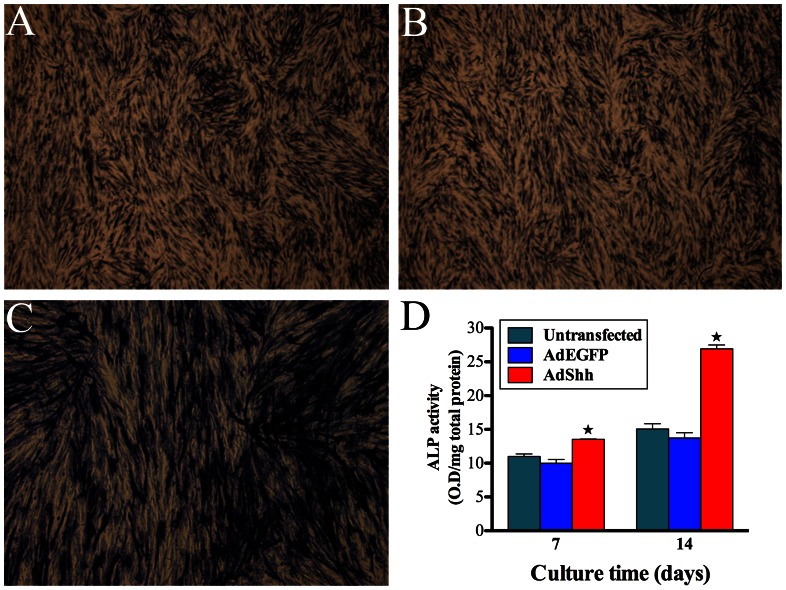
ALP activity analysis. ALP staining in untransfected hDPCs (A), AdEGFP-transfected hDPCs (B), AdShh-transfected hDPCs (C) at 14 days after gene transduction. (D) The ALP activity of untransfected, AdEGFP-transfected and AdShh-transfected hDPCs was measured with the pNPP assay at days 7, and 14 after gene transduction. (A, B, C, ×50). (n = 3 for each group, ^★^indicates significant differences between AdShh-transfected vs. untransfected and AdEGFP-transfected, *p*<0.05).

As shown in fig. 2A, B, C, ALP staining was more intense for AdShh-transfected hDPCs than AdEGFP-transfected and untransfected cells. Meanwhile, quantitative assay data showed that ALP activity in AdShh-transfected hDPCs was higher than that of either AdEGFP-transfected or untransfected cells at days 7, and 14 (*p*<0.05), with no significant difference between AdEGFP-transfected and untransfected cells (fig. 2D).

**Figure 3:**
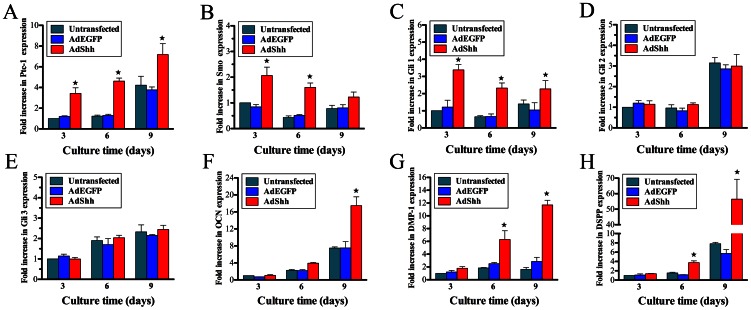
Odontoblastic differentiation analysis. Real-time PCR analysis of odontoblastic differentiation of untransfected, AdEGFP-transfected and AdShh-transfected hDPCs at days 3, 6, and 9, respectively. (A) Ptc-1; (B) Smo; (C) Gli 1; (D) Gli 2; (E) Gli 3; (F) OCN; (G) DMP-1; (H) DSPP. (n = 3 for each group, ^★^indicates significant differences between AdShh-transfected vs. untransfected and AdEGFP-transfected, *p*<0.05).

Compared with AdEGFP-transfected and untransfected hDPCs, up-regulation in the Hedgehog target genes Ptc-1, Smo, and Gli 1 was achieved for AdShh-transfected hDPCs, with significant difference at days 3, 6, and 9 for Ptc-1 and Gli 1, and at days 3, and 6 for Smo, while there was no significant change for Gli 2 and Gli 3 (fig. 3A, B, C, D, E). Importantly, the expression of OCN for hDPCs transduced with AdShh was significantly increased compared with AdEGFP-transfected or untransfected group at day 9 (*p*<0.05) (fig. 3F). Moreover, the enhanced expression of DMP-1, DSPP was observed in AdShh-transfected hDPCs as compared with AdEGFP-transfected or untransfected cells at day 6, even became more pronounced when the culture time was extended to day 9 (*p*<0.05) (fig. 3G, H). There was no significant difference for Ptc-1, Smo, Gli 1, Gli 2, Gli 3, OCN, DMP-1, and DSPP between AdEGFP-transfected and untransfected hDPCs.

### Cyclopamine treatment analysis

**Figure 4:**
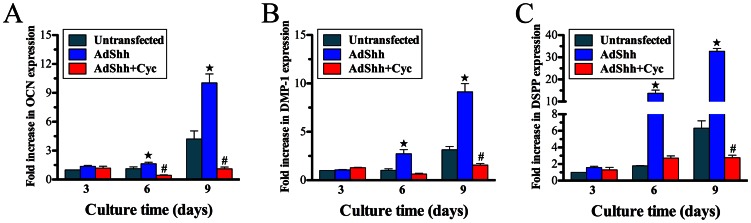
Cyclopamine treatment analysis. Real-time PCR analysis of odontoblastic differentiation of AdShh-transfected hDPCs in the presence of cyclopamine (AdShh+Cyc) at days 3, 6, and 9. (A) OCN; (B) DMP-1; (C) DSPP. (n = 3 for each group, ^★^indicates significant differences between AdShh+Cyc vs. AdShh-transfected, *p*<0.05, **^#^**indicates significant differences between AdShh+Cyc vs. untransfected, *p*<0.05).

The results of real-time PCR analysis showed that odontoblastic differentiation of AdShh-transfected hDPCs was repressed in the presence of Cyc, with significant difference at days 6, and 9 for OCN, DMP-1, and DSPP as compared with AdShh-transfected hDPCs (*p*<0.05) (fig. 4). Besides, the gene expression of OCN at days 6, and 9, as well as DMP-1, DSPP at day 9 for AdShh-transfected hDPCs treated with Cyc, was significantly lower than that of untransfected hDPCs, respectively (*p*<0.05) (fig. 4).

### Cell adhesion and growth assay

**Figure 5:**
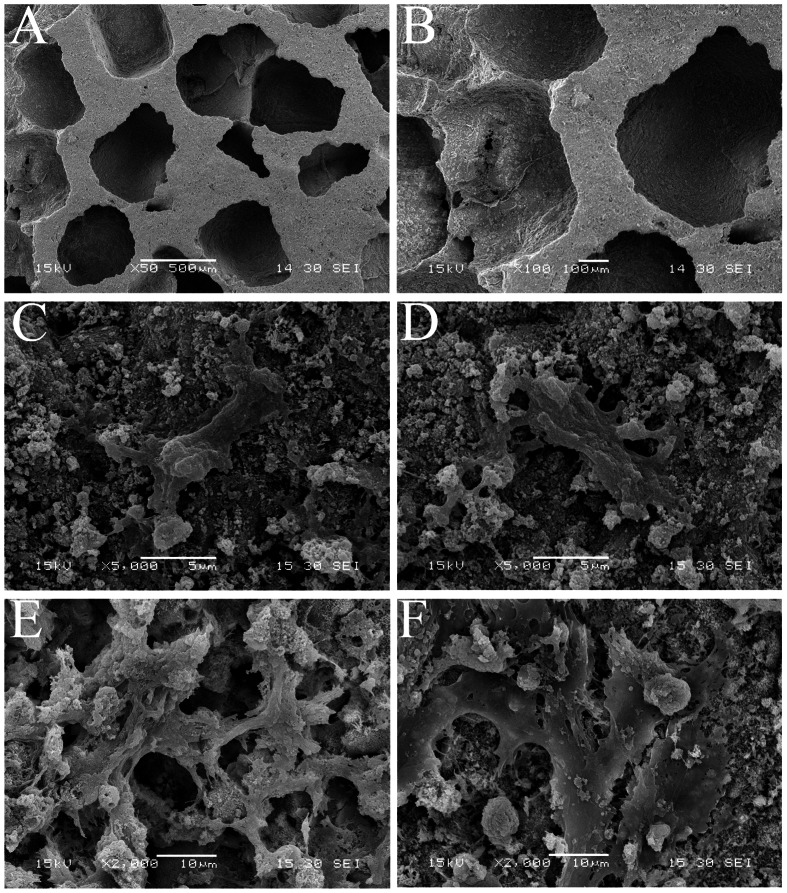
SEM analysis. SEM evaluation of porous CPC showing an average pore diameter of 300–500 µm (A, B). After cultured for 1 day, untransfected (C) and AdShh-transfected hDPCs (D) were attached and spread well on the surface of two scaffolds. At day 4, untransfected (E) and AdShh-transfected (F) hDPCs grew well and reached approximately confluence. (A, ×50; B, ×100; C–D, ×5000; E–F, ×2000).

Cell attachment and growth of AdShh-transfected and untransfected hDPCs seeded on porous CPC scaffolds were examined by SEM (fig. 5). As shown in fig. 5A, B, CPC scaffold utilized in present study, had an average pore diameter of 300–500 µm in size, and interconnection pores of 250 µm. More importantly, after cultured for 1 day, both untransfected and AdShh-transfected hDPCs were attached and spread well on the surface of CPC scaffolds, respectively (fig. 5C, D). When the culture time was extended to 4 days, these two kinds of cells grew well and reached approximately confluence on scaffolds (fig. 5E, F). All these data demonstrated that it had no obvious cytotoxicity by gene transduction. It also suggested that the CPC scaffold was suitable for the following study in vivo, with the advantage of facilitating hDPCs adhesion and growth onto its surface.

### Ectopic mineralized tissue formation analysis

**Figure 6:**
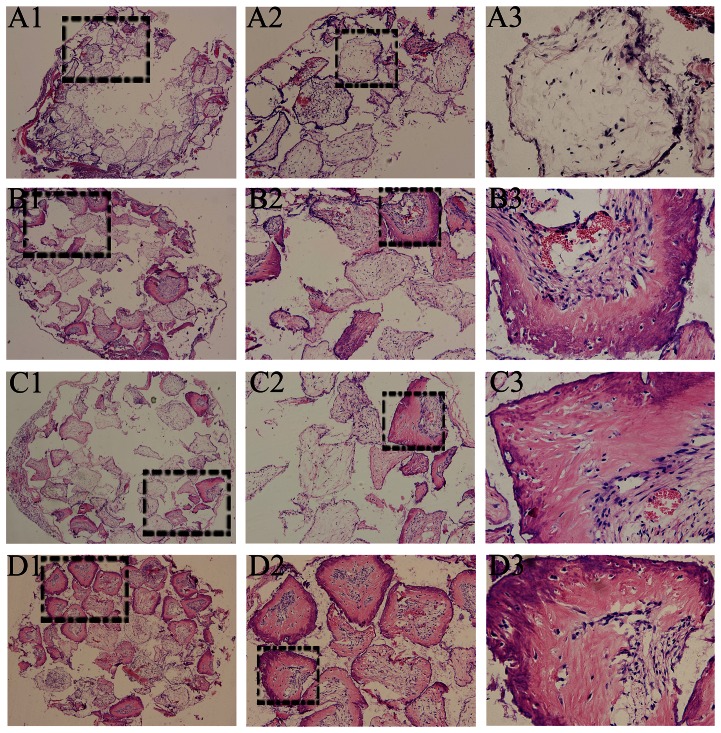
Histological findings at 8 weeks. The mineralized tissue was found in group B, (untransfected hDPCs/CPC complexes) (B1–B3), group C (AdEGFP-transfectd hDPCs/CPC complexes) (C1–C3), and group D (AdShh-transfected hDPCs/CPC complexes) (D1–D3), while there was no mineralize tissue formation in group A. (CPC alone) (A1–A3). (A1–D1, ×40; A2–D2, ×100; A3–D3, ×400).

**Figure 7:**
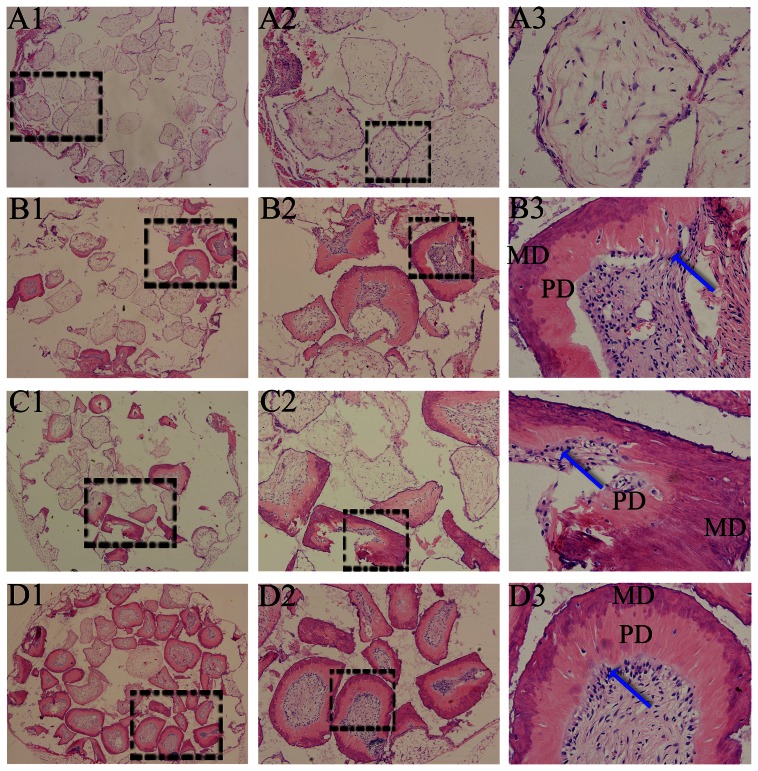
Histological findings at 12 weeks. The images of (A1–A3), (B1–B3), (C1–C3), (D1–D3) represent groups A, B, C, D, respectively. Dentin-like tissue (MD: mineralized dentin; PD: predentin), even elongated, polarized, and arranged odontoblast-like cells (blue arrow) were observed in group B, C and D. (A1–D1, ×40; A2–D2, ×100; A3–D3, ×400).

The mineralized tissue formation was observed in all groups, except CPC alone group (group A) at either week 8 or week 12 time point. At week 8, a small amount of mineralized tissue was found in group B (untransfected hDPCs/CPC complexes), and group C (AdEGFP-transfected hDPCs/CPC complexes), while there was more mineralized tissue formation in group D (AdShh-transfected hDPCs/CPC complexes) (fig. 6). When the implantation time was extended to 12 weeks, there was more quantity and mature mineralized tissue formation in all three groups as compared with that for week 8 time point, while the most mineralized tissue was also found in group D. Interestingly, the structures similar to dentin-pulp complex were observed in all three groups, including mineralized dentin-like tissue stained deep red in the outside layer, and predentin-like tissue stained lighter in the middle layer. Importantly, a large number of cells even elongated, polarized, and arranged odontoblast-like cells near to the middle layer, were distributed in the inner layer (fig. 7).

**Figure 8:**
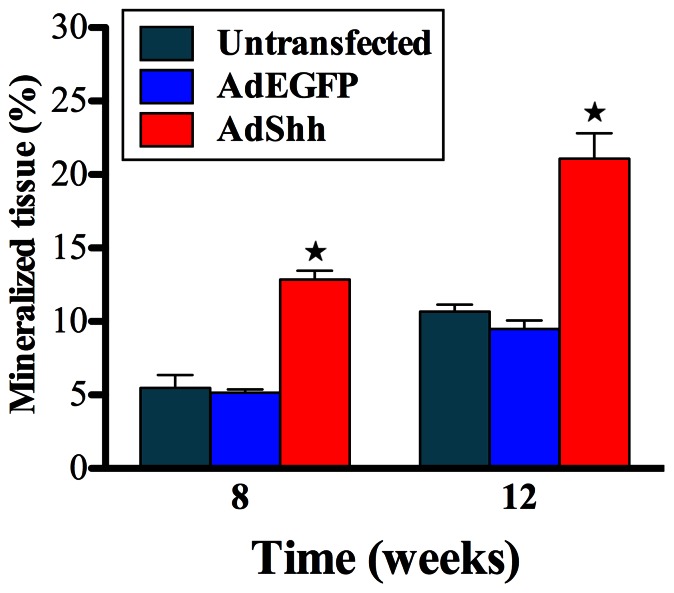
Histomorphometric analysis. Mineralized tissue area of the implants among the three groups was assessed at weeks 8, and 12 after implantation. (n = 6 for each group, ^★^indicates significant differences between AdShh-transfected vs. untransfected and AdEGFP-transfected, *p*<0.05).

Histomorphometry analysis showed significantly more mineralized tissue formation has been observed in group D (12.87±1.18% and 21.09±4.71%, respectively) than group B (5.47±1.77% and 10.68±0.94%, respectively) and group C (5.15±0.45% and 9.49±1.17%, respectively) at both 8 and 12 week time points (*p*<0.05). Besides, there was no significant difference between group B and group C (fig. 8).

### Immunohistochemistry analysis

**Figure 9:**
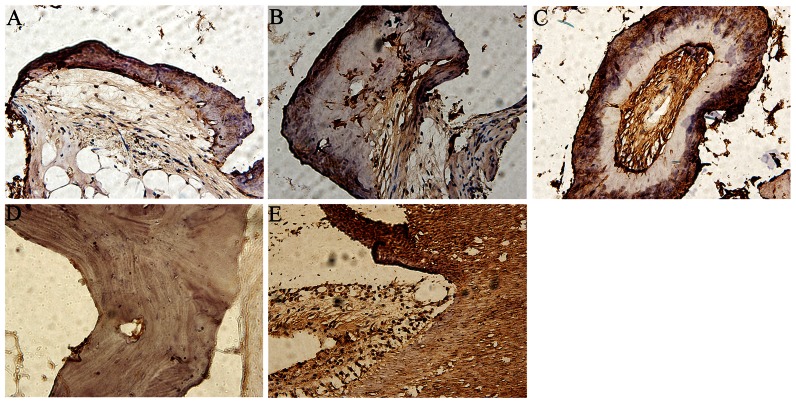
Immunohistochemistry analysis. Immunohistochemistry assay for DSPP protein on the samples for group B (A), group C (B), and group D (C) at 12 week time point. The samples of human bone (D) and tooth (E) tissues were treated as control groups. (×400).

As shown in fig. 9, the samples of group B, group C, and group D, showed positive staining of DSPP protein in extracellular matrices of mineralized dentin-like tissue, and the cytoplasm of cells distributed in the inner layer. Moreover, there was much stronger positive staining for group D. The similar positive staining of DSPP protein was also seen in the sample of human tooth tissue, conversely, the sample of human bone tissue presented almost negative staining.

## Discussion

The purpose of present study was to evaluate the effect of Shh on proliferation and odontoblastic differentiation of hDPCs as well as underlying signal pathway mechanism. Importantly, the potential for mineralized tissue regeneration by AdShh transfected hDPCs and porous CPC was also investigated in nude subcutaneous ectopic implant models.

Previous studies have demonstrated that DPCs could be induced to express odontoblastic characteristic under differentiation-inducing reagents dexamethasone (Dex), β-glycerophosphate (β-Gly) and ascorbic acid. Importantly, growth/transcription factors could further enhance the expression of odontoblastic marker genes [22]–[24]. Shh, as a member of the vertebrate Hedgehog family, has been reported to be characteristically expressed at each stage of tooth development, including in the presumptive tooth-forming area at the epithelial thickening stage, enamel knot at the early cap stage and the inner enamel epithelium at the late cap and bell stages [10]. Recent reports have showed that Shh was critical for odontoblast and ameloblast differentiation [25]–[26]. However, whether Shh could promote odontoblastic differentiation of DPCs via ex vivo gene therapy, and combine with a suitable scaffold for dental tissue regeneration was far from clear.

Adenoviral delivery system may have the potential to provide more sustained protein production and to deliver proteins in a more physiologic manner than exogenous protein release. Moreover, it has been suggested that endogenously synthesized proteins have greater biologic activities than exogenously administered recombinant proteins [20], [27]–[29]. In present study, adenoviral transfer efficiency reached about 60–80% without obvious cell death. Importantly, the transient overexpression of Shh for about two weeks could induce a mineralized tissue-formation cascade but avoid side effects that may occur in other longer-term expression system such as retrovirus delivery system.

CPC is a bioactive material that is available in powder and liquid forms, which, when mixed, primarily form hydroxyapatite [30]. Self-setting CPC material has been widely used for bone regeneration due to its potential to stimulate osteogenesis. Their excellent mechanical properties and biocompatibility properties suggest that CPC is superior to pure calcium hydroxide (CH) material. Recent study has reported that CPC could promote the formation of mineralized nodules, the induction of ALP activity in the early stage, and the up-regulation of odontoblastic expression [16]. Analysis of these results suggested that CPC material could facilitate the growth and differentiation of hDPCs, which might act as a scaffold combination with hDPCs for dental tissue regeneration.

The proliferation and odontoblastic differentiation of AdShh-transfected hDPCs were systematically evaluated in present study. Previous studies have demonstrated that Shh could promote proliferation of bone marrow-derived mesenchymal stem cells (BMMSCs) and periodontal ligament stem cells (PDLSCs) [31]–[32]. Similarly, present study also showed AdShh-transfected hDPCs possessed greater proliferation capacity, which might be benefit for dental tissue regeneration in vivo. ALP, OCN, DSPP and DMP-1 were chosen in present study as the markers for odontoblastic differentiation. ALP has been used as an early standard parameter to monitor cell differentiation, it can regulate organic and inorganic phosphate metabolism and act as a plasma membrane transporter for inorganic phosphates [33]. OCN, as a later marker of cell differentiation, is related to matrix deposition and mineralization [34]. More importantly, as the major dentinal non-collagenous proteins, DSPP is essential for dentin mineralization, while DMP-1 is also specific for dentin and is considered as a candidate gene for dentinogenesis imperfecta [7], [35]–[36]. The higher level of ALP activity and enhanced expression of OCN, DMP-1, and DSPP, indicated that Shh could promote odontoblastic differentiation of hDPCs.

In present study, underlying signal pathway of Shh on hDPCs was also preliminarily evaluated in vitro. Up-regulation in the expression of Ptc-1, Smo, and Gli 1 was observed on AdShh-transfected hDPCs suggested that the enhanced effect of Shh on hDPCs might relate to Shh/Gli 1 signaling pathway. It was initiated by the binding of the Hedgehog ligand to its transmembrane receptor Ptc1, relieving suppression of the transmembrane protein Smo followed by activating an intracellular cascade that results in activation of Gli 1. This signaling pathway has been reported to play an important role in regulation of the cell cycle, direction of cell differentiation, and alteration of cell survival [37]–[39]. Theoretically, it may be a feasible strategy to promote proliferation and differentiation of DPCs via stimulating Shh/Gli 1 signaling pathway. In order to further confirm the role of Shh/Gli 1 signaling pathway in this process, the odontoblastic differentiation of AdShh-transfected hDPCs was measured in the presence of Cyclopamine (Cyc), which is a potent Shh/Gli 1 signaling pathway antagonist targeting the SMO protein. The repressed gene expression of OCN, DMP-1, and DSPP verified that Shh promoted odontoblastic differentiation of hDPCs via Shh/Gli 1 signaling pathway.

Histology and immunohistochemistry analyses were processed to evaluate mineralized tissue formation of AdShh-transfected hDPCs combination with porous CPC. Throughout the whole observation period, mineralized tissue formation could be found in either untransfected or AdEGFP-transfected hDPCs/CPC complexes, while there was more mineralized tissue formation in AdShh-transfected hDPCs/CPC complexes. Furthermore, the mineralized tissue possessed the appearance of dentin-like features such as the structures similar to dentin-pulp complex including mineralized dentin, predentin, and odontoblast-like cells. Besides, the dentin-like tissue and the cells distributed in the inner layer showed strong positive staining for DSPP, which was similar to the sample of human tooth tissue. The results of cell/tissue morphology and DSPP expression suggest that the mineralized tissue more resembled dentin tissue in present study [40]. Previous study has showed that the combination of BMP-2-transfected pulp stem cells and hydroxyapatite/tricalcium phosphate scaffold just formed bone-like tissue without any dentin-like features [7]. We believe that porous CPC used in present study may be a more appropriate scaffold for dental tissue regeneration, while combining with Shh could further induce the dentin-like mineralized tissue formation. However, larger animal models with in situ placement, as well as a longer observation time, need to be investigated in future to provide more clinically relevant data.

In conclusion, Shh could enhance proliferation and odontoblastic differentiation of hDPCs, while Shh/Gli 1 signaling pathway might play an important role in this process. Moreover, more dentin-like mineralized tissue was achieved in combination with AdShh-transfected hDPCs and porous CPC in vivo, which might be a better alternative for dental tissue regeneration.

## Supporting Information

Table S1 **A series of data, including the gene names, accession numbers, primer sequences, and product size, is listed.**
(TIF)
