# Supplementary figures and images for: Enhanced Dentin-Like Mineralized Tissue Formation by AdShh-Transfected Human Dental Pulp Cells and Porous Calcium Phosphate Cement
Source: PLoS One. 2013 May 10;8(5):e62645. doi: 10.1371/journal.pone.0062645 (PMC3651081; doi:10.1371/journal.pone.0062645)

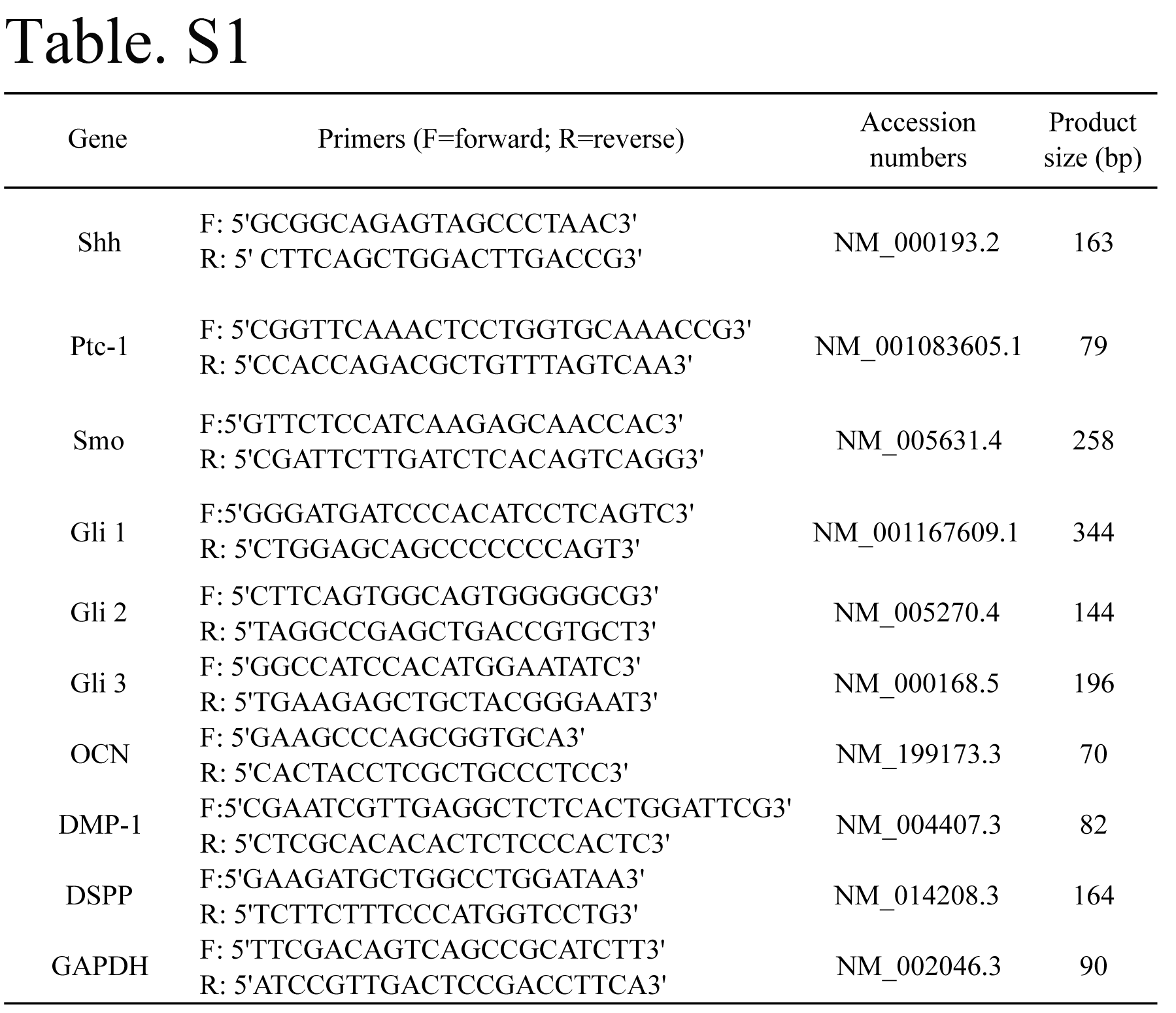

Supplement: Table S1 — A series of data, including the gene names, accession numbers, primer sequences, and product size, is listed. (TIF) [file pone.0062645.s001.tif]
